# Medical Opinion and Movement

**Published:** 1910-10-01

**Authors:** 


### Medical Opinion and Movement.

### Chlorosis.

In an interesting article in the *Münchener Medizinische Wochenschrift* Dr. P. Moravitz puts forward the view that not infrequently young girls may present all the typical signs of chlorosis in spite of the blood showing a normal or almost normal content in hæmoglobin. He states that in the last six months he has had under examination in his clinic thirty-six patients who showed the classic symptoms of chlorosis without any notable diminution of hæmoglobin in the blood. In eighteen of these patients he has obtained in a short time a cure or considerable improvement by administration of iron preparations, so that they yield to the same treatment as those suffering from chlorosis with well-marked anæmia. He concludes from these observations that the anæmia, instead of constituting the cardinal symptom of chlorosis is only one of its manifestations, and that menstrual troubles, the *bruit de diable* and œdema, as well as the subjective symptoms to which chlorotics are subject are not always dependent upon the anæmia. He suggests that the curative effect of iron is not due to its stimulating effect upon the hæmatopoiëtic organs, as is evidenced by the fact that, contrary to arsenic, it is useless in the non-chlorotic anæmias.

### Quinine and Urea Anæsthesia.

Dr. Thibault first drew attention to the analgesic effects of injections of chlorhydrate of quinine and urea some two or three years ago. Dr. W. O. Green, of Louisville, reports four cases in which he has successfully used this preparation for carrying out operations on the anal region. The operation in each case consisted in the removal of hæmorrhoids, and the great advantage claimed for this analgesic is that the effect persists for several days, thus relieving the patient from the pain and discomfort that almost invariably follow these operations. The author used a 1-per-cent. solution and injected 50 to 80 minims into the tissues around the anus. In one case the anæsthesia was incomplete and only persisted for twenty-four hours, but in another case it lasted for four days, and in the two others seven and nine days respectively. In order to obtain complete analgesia the tissues must be thoroughly distended. In three out of the four cases a certain amount of induration resulted which disappeared in a few days.

### A Rare Case of Aerophagy.

An extraordinary case of aerophagy was reported by Drs. Schlesinger and Von Friedländer at a meeting of the Vienna Medical Society. The patient, a man, had suffered for seven years from severe attacks of gastric pain, for which laparatomy had been performed four times without the discovery of any lesion in the abdominal viscera and without obtaining any beneficial result. The pains were so intense as to necessitate the administration of morphia or chloroform. He had undergone nearly a thousand anæsthetic administrations and was accustomed to such doses of morphia and scopolamine as would be generally considered fatal. During the attacks the stomach assumed the appearance of a hard elastic tumour, giving a tympanitic note on percussion. It was distended with gas, which could not be evacuated by passage of a sound. During anæsthesia, the gas was evacuated by the mouth, and the patient was soon relieved. Careful observation failed to discover that the patient had the habit of swallowing air. Dr. Von Friedländer performed gastrostomy, and found that the stomach was small, mobile, and not hypertrophied. He made a permanent gastric fistula, into which he fixed a catheter fitted with a tap, which the patient opens as soon as he feels any discomfort, so as to evacuate the gas. Since the operation the attacks have not recurred.

### The Prevalence of Hernia.

In the thirty-eighth annual report of the chief medical officer of the Local Government Board there are several researches included which are of medical or surgical interest. One of these is a report on hernia by Dr. Basil Cook. Discussing the influence of occupation upon this defect, he notes that in such laborious employment as those of stokers, coal-heavers, etc., the average incidence of hernia in the early years of manhood is very high, whereas after a certain age—twenty-five for coalheavers, thirty-five for gas-works stokers, for instance—the incidence is actually less than normal. In sedentary occupations such as commercial clerkships, on the contrary, the " trade ratio " is less than the standard up to the age of thirty-five, but exceeds the normal in a rapidly increasing degree in later years. The explanation of this seeming paradox is that in the laborious occupations all those with any inherent predisposition to hernia are weeded out during the first few years, and those that remain are the exceptionally powerful; thus coalheavers over sixty-five suffer from hernia only 39 per cent. of the extent to which all workers taken together have it at that age. Among clerks, on the other hand, the hernia figures in middle age are swelled by the inclusion of those who have contracted hernia in some more arduous walk of life, and are no longer able to undertake any but sedentary employment. It is a curious fact that the proportion of hernia in the general population increases steadily with increasing age up to sixty-five, after which it recedes; but with advancing age there is always a tendency for a single hernia to become double. In the services it is an established fact that a material gain to the nation in military and naval efficiency has been secured by the increasing number of cures through radical operation. Among civilians the attitude of those who have had a recurrence after operation for radical cure has probably led to under-statements as to the percentage of recurrences.

### Pneumonia in Young Children.

The verdict of a searching inquiry into the subject of infantile pneumonia is stated by Mr. Foulerton in the same report. After eliminating as far as possible all confusing elements, he finds that the principal conditions of disease, apart from those of antenatal origin, which are especially associated with fatal pneumonia during the first two years of life, are themselves amongst the effects of artificial feeding. These conditions, so far as they influence the mortality from pneumonia, operate in the main by causing a state of general enfeeblement in which the power of resistance against bacterial invasion of the lungs is lowered. The social conditions which unfavourably affect the mortality are essentially those which cause also a high death-rate from infantile diarrhœa—the overcrowding of land with houses, and of houses with persons. Poverty in itself is not an essential determining cause of this mortality; for the incidence of infantile diarrhœa and pneumonia in areas of industrial prosperity is sufficiently marked. It does not appear that any special administrative measures for the reduction of this mortality are necessary or practicable, beyond such as are already operative or contemplated to promote the natural feeding of children, to check the spread of epidemic diseases, and to provide for the satisfactory housing of the working classes.

### Hot Packs for Chorea Gravis.

The rarity of chorea gravis of late years is a striking fact which seems to have impressed a good many physicians whose memory takes them back some decades. Dr. W. Pasteur ascribes it to the introduction of salicylates in the treatment of acute rheumatism. Nevertheless cases of intractable chorea do occur, and an especially severe one is recorded by this physician in the *Archives of the Middlesex Hospital*. A boy of seventeen was admitted for well-marked, but not excessive, choreic movements; there was no rheumatic history, personal or familial. For a fortnight he was treated with chloretone, chloral, and bromides, in liberal dosage. However, not only did he not improve but he got worse. Ultimately he began to vomit all his food, secrete almost no urine, waste rapidly, and become gravely exhausted; the movements were so severe that morphia and chloroform controlled them but temporarily. In this serious condition Dr. Pasteur bethought him of the hot pack. This was given with great difficulty, and the boy fell asleep before being removed from it, and slept for eight hours. The renal function was promptly restored, and improvement was immediate in other ways. After a week he began to lose ground again, and packs were again resorted to. In six days he had nine hot packs, after which convalescence was rapid and uninterrupted. After the first pack no drugs were given except cod-liver oil and iron. In these cases of severe and dangerous chorea, Dr. Pasteur says the hot pack should be tried first in preference to any other form of treatment, though sedatives may also be given if desired.

### The Gonococcus and Trachoma Bodies.

According to Herzog, who writes in the *Deutsche Med. Woch.*, the so-called trachoma bodies, or chlamydozoa, are but involution forms of the gonococcus. He has by means of prolonged cultivation of a pure culture of the gonococcus been able to produce bodies morphologically identical with chlamydozoa. He has found typical trachoma bodies in the eye-secretions of undoubted cases of gonorrhœal ophthalmia after the acute stages of the disease had passed off. In new cases of trachoma he has noticed intra-cellular organisms, which morphologically would seem to lie between the trachoma body and the ordinary gonococcus. He has even had the courage to inoculate the blind glaucomatous eye of a man with a pure fresh culture of the gonococcus, and within fourteen days has isolated typical trachoma bodies from the patient's conjunctival secretion. It is not possible to say that this work definitely settles the question of trachoma bodies, but it must inevitably stimulate research with the object of solving the problem.

### Familial Hæmolytic Icterus.

Attention has lately been called by a number of French observers to a condition of familial icterus associated with a large spleen and a tendency to hæmolysis. As a rule, acute febrile paroxysms are present from time to time. Blood examination shows marked weakness of the erythrocytes in such cases. Aschenheim, who writes in the *Münchener Med. Woch.*, states that German observers do not seem to have come across the condition to a great extent. He describes a case of chronic jaundice with urobilin in the urine, bile in the stools, enlargement of liver and spleen. The condition occurred in two members of a family, father and son. Blood examination gave results similar to those obtained in the French cases. The author would classify such a condition under the general term "hæmolytic icterus," but is not convinced that it is necessarily of a familial character. He points out that it is necessary to distinguish such a condition from one resulting from splenic leukæmia, Banti's disease, etc. The jaundice alone is not sufficient for differential diagnosis, since this may be the result of other maladies. It would, however, be an easy matter to distinguish hæmolytic icterus from typical cases of other diseases, though when causes of symptoms are not well marked some difficulty in diagnosis may occur.

### Menstrual Hyperæmia of the Liver.

The frequency of hepatic affections in women and the well-known fact that hepatic colic often occurs during menstrual periods, presupposes some definite relation between these pathological conditions and the utero-ovarian functions, the menses in particular. Professor Chvostek, in the *Vratchebnaia Gazeta*, reports the results of his researches into this question. He has been able to show by percussion of the liver during the menstrual periods that in many cases there is a considerable enlargement of that organ at this time, amounting occasionally to an increase of as much as two fingers' breadth in the liver-dulness. This enlargement gradually diminishes with cessation of the flow, but the organ takes a few days longer to regain its normal proportions. The phenomenon is to be found in normal patients as well as in those who are subject to hepatic disorders. The author believes menstrual tumefaction to be dependent on an active hyperæmia resulting from internal secretion of the ovaries. This periodic flux of blood explains the more or less frequent apparition of hepatic affections during menstruation and pregnancy, as well as the aggravation of symptoms at such times in cases where hepatic mischief already exists.

### Absence of Uterus.

At the annual meeting of the American Gynæcological Society, Dr. T. S. Cullen, of Baltimore, described an extraordinary case of urogenital malformation and malposition. The patient, a girl of seventeen, gave a history of never having menstruated. On examination the vagina was found to be totally absent, but per rectum a mass could be felt filling the right half of the pelvis, which at first was taken for a uterus with retained menstrual flow. At the operation an incision was made between the bladder and rectum until the mass was felt. This was found to be hard. The abdomen was at once opened and an irregular tumour noticed in the right half of the pelvis, which, by reason of a cleft on its inner aspect, was thought to be a kidney. Examination of the right renal pocket showed that no kidney was to be found there, nor did one exist on the left side either. Thus the case was one of right pelvic kidney. No uterus existed, but half of the right Fallopian tube was seen emerging from the inguinal canal. The remaining portion of the tube and its ovary lay in the canal. A small loop of round ligament was discernible.

### Iodine Tincture and Gangrene.

Much has of late been written concerning the virtues of tincture of iodine as a skin antiseptic, both in preparing the cuticle for operation and in cleaning up wounds. The disadvantages of its use are not mentioned, however, to any great extent, so that it is well that attention should be called to two cases in which its employment was followed by gangrene of the skin. Dr. Hinderberg, in the *Münch. Med. Woch.*, reports that he had treated two labourers who came to him with wounds of the toes caused by the axe slipping while cutting wood. They were seen within a few moments of the accident, the bleeding wounds being painted freely with tincture of iodine and an aseptic bandage applied. Two days later, on removing the latter, the edges of the wounds were found to be red and inflamed, and in three days after that all the phenomena of gangrene were present. This fortunately was entirely superficial, and under treatment quickly cleared up. The author explains the occurrence of this condition by supposing that the application of the drug to the bleeding wounds allowed of a sufficiently large quantity of it to be absorbed by the gaping vessels in the walls of the wound to induce gangrene. He therefore advises that in similar cases the edges of the wound be carefully protected by iodoform gauze before swabbing its depths and the neighbouring parts with the drug.

### Successful Operation for Sarcoma of Brain.

There is frequently a tendency to belittle the progress which neurology has made in the last twenty years, on the ground that it is all very well to be able to diagnose cases with greater certainty, but that it will be time enough to talk when they can be cured. That even the most apparently hopeless cases can be undertaken with possibility of success, even though a faint one, is well shown by the case reported in the *Edinburgh Medical Journal* by Mr. Hodsdon. In February 1908 he operated upon a man diagnosed as the subject of a cerebral tumour of the frontal lobe. The patient had exhibited symptoms for about two months; he had, amongst other troubles, advanced double optic neuritis. At the operation a large tumour was exposed, and was removed with some of the surrounding brain substance. On microscopical examination it was proved to be a sarcoma. About a month after operation the patient was able to go home, though he had a large hernia cerebri. His sight was not improved, but his intense headaches were relieved, and vomiting had also ceased. In June 1910 this man reported himself for inspection; he has now no hernia cerebri, and, indeed, the scar of the incision is barely visible. He feels perfectly well, though vision is still confined to the appreciation of light from darkness. This remarkable case was well worthy of publication.

### Serum Diagnosis of Pregnancy.

The newest development of serum diagnosis would seem to be the determination of pregnancy by this method. Fieux and Mauriac publish in the *Annales de Gynécol. et d'Obstetr.* some researches which reveal to them facts whereon the serum diagnosis of pregnancy may be based. The intoxication of the mother by absorption of products from the syncytium of the chorionic villi is a conception familiar to those who study the toxæmias of pregnancy; and it is assumed that an antibody would naturally be produced to neutralise it. The specific reaction is sought on lines essentially similar to those of the Wassermann reaction, and the conclusions drawn from extended experiments are summarised by the authors thus: The maternal blood contains during the early period of pregnancy an antibody which is specific and formed as a defence against the toxic element of the young chorionic villi. This specific antibody is present in most abundance during the second and third months of pregnancy; it diminishes rapidly after that time, and is quite impossible to discover in the later months of gestation. It occasionally persists for a short time after an early abortion. These facts render possible the serum diagnosis of pregnancy during the second and third months—that is, just during the period when the diagnosis by other methods is least certain and reliable, and most required.

